# Multicenter performance evaluation of the Alinity m CMV assay for quantifying cytomegalovirus DNA in plasma samples

**DOI:** 10.1128/jcm.00415-23

**Published:** 2023-09-20

**Authors:** Miae Lee, Eliseo Albert, Els Wessels, Soo-Kyung Kim, Hae-Sun Chung, Estela Giménez, Tom Vreeswijk, Eric C. J. Claas, Yan Chin Tai, Birgit Reinhardt, Mark M. Sasaki, David Navarro

**Affiliations:** 1 Ewha Womans University College of Medicine, Seoul, Republic of Korea; 2 Hospital Clinico Universitario de Valencia, Valencia, Spain; 3 Leiden University Medical Center, Leiden, the Netherlands; 4 Abbott Laboratories (Singapore) Pte. Ltd., Singapore; 5 Abbott GmbH, Wiesbaden, Germany; 6 Abbott Molecular Inc., Des Plaines, Illinois, USA; Mayo Clinic, Rochester, Minnesota, USA

**Keywords:** nucleic acid amplification test, DNA, immunocompromised, transplantation, high-throughput diagnostic assay

## Abstract

Monitoring of cytomegalovirus (CMV) viral load is critical for informing treatment decisions in order to prevent the severe health consequences of CMV infection or reactivation of latent CMV in immunocompromised individuals. This first field evaluation examined the analytical and clinical performance of the Alinity m CMV assay. Analytical performance was assessed with a commercially available six-member panel, while the clinical performance evaluation compared the Alinity m CMV assay to the RealTi*m*e CMV assay and a laboratory-developed test (LDT) as the test of record at three large hospital-based clinical laboratories. Precision of the Alinity m CMV assay was demonstrated with total standard deviation (SD) between 0.08 and 0.28 Log IU/mL. A total of 457 plasma specimens were tested on the Alinity m CMV assay and compared to the test of record at each site (*n* = 304 with RealTi*m*e CMV and *n* = 153 with LDT CMV). The Alinity m CMV assay had excellent correlation (correlation coefficient *r* ≥0.942) in comparison to the RealTi*m*e CMV or LDT CMV assays. The mean observed bias ranged from −0.03 to 0.34 Log IU/mL. Median onboard turnaround time of Alinity m CMV was less than 3 h. When the CMV assay is run on the Alinity m system, it has the capacity to shorten time to result and, therefore, to therapy.

## INTRODUCTION

Cytomegalovirus (CMV) is a member of the herpesvirus family with a high seroprevalence worldwide (1). Primary infection can cause mild or subclinical disease in immunocompetent individuals and establishes life-long latency (2). Primary infection or reactivation of latent CMV has severe and life-threatening consequences in newborns and in patients who are immunocompromised, including the elderly, patients with AIDS, and stem cell and solid organ transplant recipients (2 – 4). Monitoring of CMV DNA load in plasma or blood of transplant recipients is critical to initiate preemptive therapy and, thus, prevent the occurrence of CMV end-organ disease (5) as CMV infection is associated with graft rejection and decreased patient survival (6).

Nucleic acid amplification tests (NAATs) have high sensitivity and specificity for target pathogens such as CMV and provide accurate quantitation to inform appropriate patient management (7). Many laboratories had established laboratory developed tests (LDTs) long time ago when commercial CMV assays were rare. However, facing a high testing burden and increased quality and documentation requirements, especially with regard to the new European *In vitro* Diagnostic Regulation, laboratories are looking for consolidating their CMV and other PCR assays on a single platform with high quality assays and low turnaround time (TAT). The Alinity m CMV assay (Abbott Molecular Inc., Des Plaines, IL, USA) (8) is a quantitative PCR assay that is run on the fully automated, continuous, random-access Alinity m analyzer with a processing capacity of 300 samples in approximately 8 h and a time to first result of <115 min. The purpose of this study was to evaluate the analytical and clinical performance of the Alinity m CMV assay and compare it to the RealTi*m*e CMV assay (9) and an LDT CMV in plasma specimens (10).

## MATERIALS AND METHODS

### Assessment of the analytical performance

The analytical precision of the Alinity m CMV assay was assessed using a six-member CMV verification panel (Exact Diagnostics, Fort Worth, TX, USA) containing non-infectious intact whole virus at 2.3–6.6 Log IU/mL in ETDA plasma. For each site, three replicates per panel member were tested over 5 days (total *n* = 45 with 15 replicates per laboratory).

Reproducibility was assessed by evaluating the performance of the assay quality controls [QCs; low positive control (LPC) and high positive control (HPC)] from multiple reagent lots across testing sites. For each site, one replicate of HPC and LPC was tested over 5 days (total *n* = 15 with five replicates per laboratory).

### Clinical performance evaluation and specimens

The performance of the Alinity m CMV assay (Abbott Molecular Inc., Des Plaines, IL, USA) was compared to two CMV assays in a multicenter, international study performed at three independent International Standard Organization accredited clinical laboratories: Ewha Womans University Mokdong Hospital, Seoul, South Korea; Hospital Clinico Universitario de Valencia, Valencia, Spain; and Leiden University Medical Center, Leiden, the Netherlands.

The study was performed in accordance with the principles of Good Clinical Practice and conducted in adherence with the Declaration of Helsinki. Only surplus remnant patient plasma specimens were used for this study. All specimens were anonymized before study initiation, and an identification number containing no patient identifiers was assigned to each remnant specimen. An approval by an ethics committee was obtained according to the institutional requirement.

Alinity m CMV assay performance was evaluated across 457 remnant clinical plasma specimens that were sent to the participating laboratories for routine CMV DNA load determination. They were collected from solid organ and hematopoietic stem cell transplant recipients and stored frozen at ≤−70°C for 0.5–6 years. The clinical specimens were retrospectively tested using the Alinity m CMV assay and the results were compared to the historical data of one of the two routine CMV assays based on real-time PCR, the RealTi*m*e CMV assay (Abbott Molecular Inc., Des Plaines, IL, USA) or an LDT CMV (Leiden, the Netherlands).

Alinity m CMV assay performance was also evaluated across the longitudinal course for 26 de-identified patients previously tested with LDT CMV. Patients were monitored between 4 and 296 days with a range of 2–10 times.

### Molecular CMV assays and workflow evaluation

The Alinity m CMV assay is a dual target assay (UL34 and UL80.5) and standardized to the first WHO International Standard (11) with a quantitative range from 1.48 to 8.00 Log IU/mL.

The RealTi*m*e CMV assay (9) is also a dual target assay (UL34 and UL80.5) and standardized to the first WHO International Standard (11). It is run on the *m*2000 platform, including automated sample preparation on the *m*2000sp and PCR on *m*2000rt. The assay has a quantitative range from 1.49 to 8.19 Log IU/mL. Its analytical and clinical performance were evaluated in prior studies (12, 13).

The LDT CMV assay is based on the previously published PCR assay utilizing primers and probes to amplify a 126-bp fragment from the CMV immediate-early antigen region, with minor modifications. DNA isolation was performed using the MagNA Pure 96 platform with the DNA and Viral NA Small Volume Kit 2.0 using the Pathogen Universal 200 protocol (Roche Diagnostics, Almere, the Netherlands). The PCR reaction was carried out using the HotStar Taq master mix (Qiagen, Hilden, Germany) in the CFX96 real-time detection system (Bio-Rad, Veenendaal, the Netherlands) (10). The LDT CMV assay has been used in routine clinical practice for nearly 20 years, is standardized to the first WHO International Standard (11), and quantifies results between 1.7 and 7.0 Log IU/mL. In clinical routine, results below 250 IU/mL are reported as <2.4 Log IU/mL since quantitation of viral loads <250 IU/mL is not considered clinically relevant in our setting.

Onboard and processing TATs of the Alinity m system were evaluated based on the automatically documented timepoints of loading samples, sample aspiration and result reporting by Alinity m.

### Statistical analysis

All analyses were performed using PC SAS (version 9.4) software (SAS, Cary, NC, USA). Relationships between quantitative variables were studied by means of Deming regression. Bland-Altman analysis was performed to evaluate the differences in quantification between the assays.

The following analysis was performed for each instrument and each panel member: The PROC MIXED procedure with the MIVQUE0 option in SAS was used to produce variance components for the model used in the analysis. The point estimates of the means and standard deviations (SDs) were reported. The SD was estimated for the within-day component, the between-day component, and the between-site component for each instrument and each panel member. All the effects were considered as random for the analyses. Any negative variance components were set to zero for these calculations. The total assay variability was defined as the sum of the within-day (residual error) component, the between-day component, and the between-site component estimates of variability. The following statistics were reported: *N*, mean, within-day SD, between-day SD, between-site SD, and total SD. For the evaluation of the quality controls, a within-day component was not included in the analysis as only one replicate of each HPC and LPC was tested per day and site.

## RESULTS

### Analytical performance

Analytical precision of the Alinity m CMV assay was assessed across the three study sites by testing a commercially available plasma CMV verification panel. The observed quantitation was similar to the expected concentrations for all panels tested with a mean bias of 0.04 Log IU/mL. SD measure of each level tested was less than or equal to 0.28 Log IU/mL (Table 1). Reproducibility was characterized by a total SD of 0.12 Log IU/mL for either LPC and HPC (Table 2).

**TABLE 1:** Precision of Alinity m CMV assay using CMV verification panels tested across three laboratories (*n* = 45 with 15 replicates per laboratory)

Panel member	*N*	Target conc. (Log IU/mL)	Mean conc. (Log IU/mL)	Difference mean-target (Log IU/mL)	Within-day component	Between-day component	Between-site component	Total
					SD (Log IU/mL)	SD (Log IU/mL)	SD (Log IU/mL)	SD (Log IU/mL)
1	45	2.30	2.33	0.03	0.28	0.00	0.00	0.28^*a*^
2	45	2.60	2.61	0.01	0.10	0.00	0.01	0.10
3	45	3.60	3.60	0.00	0.07	0.01	0.04	0.08
4	45	4.60	4.62	0.02	0.05	0.00	0.06	0.08
5	45	5.60	5.67	0.07	0.04	0.00	0.07	0.08
6	45	6.60	6.72	0.12	0.06	0.02	0.09	0.11

^*a*^ One replicate of panel member 1 was an outlier. If this outlier was removed from analysis, SD would be 0.17 Log IU/mL.

**TABLE 2:** Reproducibility of testing Alinity m CMV quality controls across three laboratories using two lots of quality controls (*n* = 15 with five replicates per laboratory)^*a*^

Control	*N*	Target conc. range (Log IU/mL)	Mean conc. (Log IU/mL)	Between-day component	Between-site component	Total
				SD (Log IU/mL)	SD (Log IU/mL)	SD (Log IU/mL)
HPC	15	5.99–6.00	6.01	0.09	0.08	0.12
LPC	15	3.14–3.30	3.24	0.04	0.11	0.12

^*a*^ HPC, high-positive control; LPC, low-positive control.

### Clinical performance

Overall, 457 plasma specimens were tested on the Alinity m CMV assay and the results were compared against one of the two comparator CMV assays: the RealTi*m*e CMV (304 specimens) or an LDT CMV assay (153 specimens).

**Fig 1:**
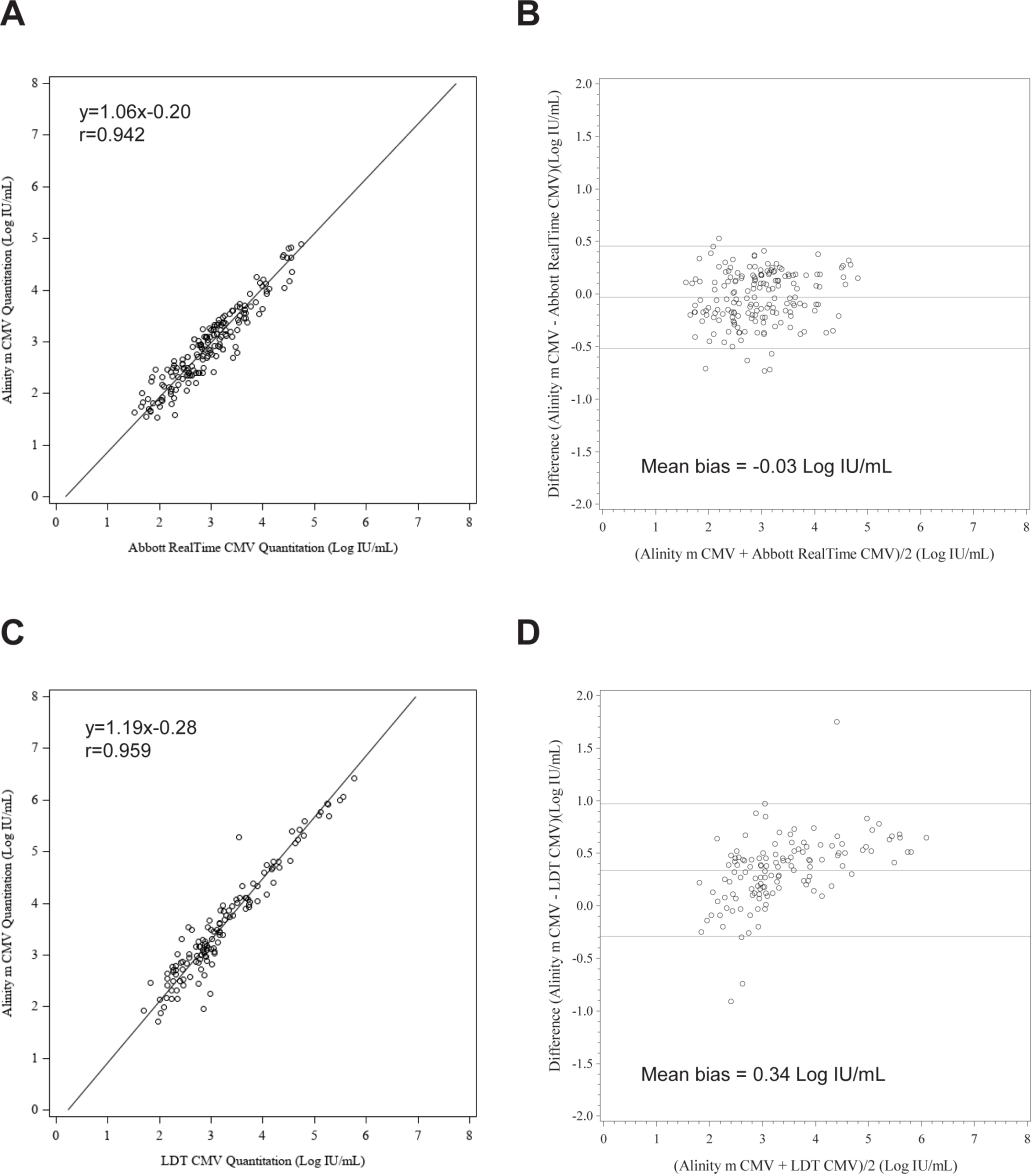
Comparison of the Alinity m CMV assay and test of record CMV assay performance with clinical plasma specimens. Deming regression of CMV levels showing correlation between the Alinity m CMV assay and (**A**) the RealTi*m*e CMV assay on *m*2000 or (**C**) an LDT CMV assay. Bland-Altman analysis showing mean bias between the Alinity m CMV assay and (**B**) the RealTi*m*e CMV assay or (**D**) LDT CMV assay. The middle line indicates the mean bias, the lines above and below indicate ±1.96 × SD.

A total of 304 remnant clinical specimens previously tested with the RealTi*m*e CMV assay were retested using the Alinity m CMV assay. The overall observed qualitative agreement, calculated as concordant negative and positive results (<LLOQ or quantitated results) between the two assays, was 89.1% (271/304) with a Cohen’s kappa value of 0.66 representing substantial agreement (14) (Table 3). Of the 304 specimens tested on the Alinity m CMV assay and the RealTi*m*e CMV assay, 174 fell within the analytical measuring range (AMR) for both assays. The correlation coefficient was 0.942 (Deming regression equation, *y* = 1.06*x* −0.20) and the mean observed bias −0.03 Log IU/mL (Bland-Altman analysis, Alinity m CMV – RealTi*m*e CMV, Fig. 1A and B).

**TABLE 3:** Alinity m CMV clinical specimen agreement of results determined by the RealTi*m*e CMV assay

		RealTi*me* CMV			
		Not detected	<LLOQ	Quantitated	Total
Alinity m CMV Assay	Not Detected	43	20	8^*a*^	71
	<LLOQ	5	25	26^*b*^	56
	Quantitated	0	3^*c*^	174	177
	Total	48	48	208	304

^*a*^ Eight specimens Not Detected by Alinity m CMV assay had a range of 1.49–1.93 Log IU/mL with the RealTi*me* CMV assay.

^*b*^ Twenty-six specimens <LLOQ by Alinity m CMV had a range of 1.52–2.16 Log IU/mL with the RealTi*m*e CMV assay.

^*c*^ Three specimens <LLOQ by RealTi*m*e CMV had a range of 1.57–1.81 Log IU/mL with the Alinity m CMV assay.

The overall observed qualitative agreement between the Alinity m CMV and the LDT CMV assays was 96.7% (148/153) with a Cohen’s kappa value of 0.87 representing almost perfect agreement (Table 4). Of the 153 specimens, 127 fell within the AMR of both assays. The correlation coefficient *r* was 0.959 (Deming regression equation, *y* = 1.19*x* –0.28) and the mean observed bias +0.34 Log IU/mL (Bland-Altman analysis, Alinity m CMV – LDT CMV, Fig. 1C and D).

**TABLE 4:** Alinity m CMV clinical specimen agreement of results determined by the LDT CMV assay

­		LDT CMV		
		Not detected	Quantitated	Total
Alinity m CMV Assay	Not Detected	21	1^*a*^	22
	<LLOQ	2	0	2
	Quantitated	2^*b*^	127	129
	Total	25	128	153

^*a*^ One specimen Not Detected by Alinity m CMV assay was quantitated at 1.81 Log IU/mL with the LDT CMV assay.

^*b*^ Two specimens Not Detected by LDT CMV assay showed 1.59 and 1.61 Log IU/mL with the Alinity m CMV assay.

**Fig 2:**
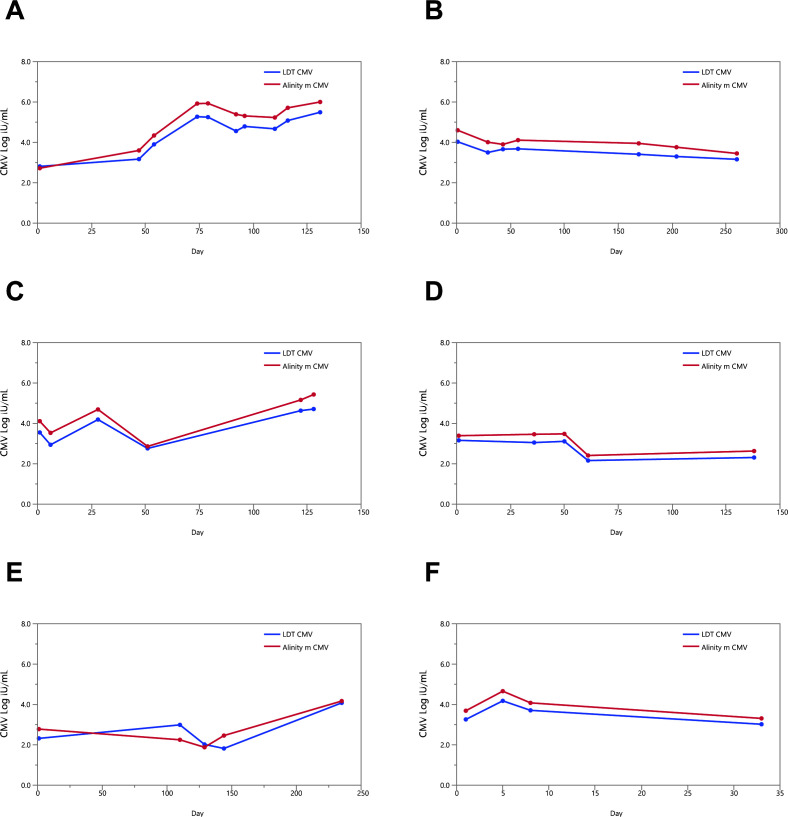
Comparison of longitudinal clinical plasma specimen results of Alinity m CMV and an LDT CMV assay. CMV viral load kinetics shown for six patients with ≥3 tests. (**A**) Patient 1 tested at days 1, 47, 54, 74, 79, 92, 96, 110, 116, and 131. (**B**) Patient 2 tested at days 1, 29, 43, 57, 169, 204, and 260. (**C**) Patient 3 tested at days 1, 6, 28, 51, 122, and 128. (**D**) Patient 4 tested at days 1, 36, 50, 61, and 138. (**E**) Patient 5 tested at days 1, 110, 129, 144, and 235. (**F**) Patient 6 tested at days 1, 5, 8, and 33.

Of the above 153 specimens, 91 tests were part of longitudinal analysis performed on 26 patients. After the baseline test, each patient underwent subsequent testing between 4 and 296 days with a range of 2–10 times. CMV viral load kinetics were very similar between Alinity m CMV and LDT CMV assays. Monitoring results for patients with at least three time points after baseline testing are shown in Fig. 2A through F.

### Workflow analysis

The Alinity m systems allowed random and continuous loading of CMV study samples side by side with samples of routine assays that were processed simultaneously on the systems. In this real-world setting, observed median onboard TAT (from placement of the specimen on the analyzer to result reporting) for the Alinity m CMV assay was 2 h 54 min across the three study sites (ranging from 2 h 7 min to 4 h 41 min). Processing TAT (from sample aspiration to result reporting) was 112–116 min with 97% of all results being reported within 114 min, across all three study sites.

## DISCUSSION

Evaluation of a CMV verification panel was performed across the three study sites using two lots of amplification reagents, three lots of lysis buffer, and three lots of sample preparation reagents. Despite testing with different instruments, operators, and reagent lots, the precision of the Alinity m CMV assay was high as shown by an SD of less than or equal to 0.28 Log IU/mL at each level tested across the AMR. One replicate of panel member 1 was an outlier which resulted in higher than expected SD. If this outlier was removed from analysis, SD would be 0.17 Log IU/mL. Unfortunately, no residual volume was left to perform any additional testing of this replicate. The observed means and total SDs for the Alinity m CMV assay HPC and LPC were 6.01 ± 0.12 and 3.24 ± 0.12 Log IU/mL, respectively.

This multicenter, international study demonstrated excellent correlation (correlation coefficient ≥0.942) between Alinity m CMV and currently used CMV assays in plasma. The Alinity m CMV assay demonstrated comparable quantitation to its predecessor, the RealTi*m*e CMV assay (bias −0.03 Log IU/mL). Overall bias between Alinity m CMV and the LDT CMV assays was 0.34 Log IU/mL, which is considered not significant in the context of biological variation and taking into account that residual stored specimens were used. This difference in quantitation could also be attributed to the intrinsic differences in the assay design such as sample extraction efficiency differences or amplicon size. Since the majority of the discordant results between the Alinity m and comparator CMV assays were at the low end of the AMR or below LLOQ of one or both assays, imprecision may also contribute to the difference in quantitation.

Of note, there was one sample with a difference of 1.75 Log IU/mL between LDT CMV and Alinity m CMV results (3.53 Log IU/mL with LDT and 5.28 Log IU/mL with Alinity m). Repeat testing with LDT CMV yielded a similar result as before (3.58 Log IU/mL), while resolution testing with the RealTi*m*e CMV assay (5.28 Log IU/mL) confirmed the Alinity m CMV assay result. Since the internal control that is present in every sample in the LDT CMV fulfills the criteria, inhibition of the sample is very unlikely. A possible explanation might be a mutation within the target region of LDT CMV, since this could impact the quantitation of this sample. The Alinity m CMV and RealTi*m*e CMV assays are dual target assays (UL34 and UL80.5), whereas the LDT CMV targets a single site in the CMV immediate-early antigen region. Another explanation could be a considerable amount of fragmented nucleic acid in this specimen that may be detected by the Alinity m CMV and RealTi*m*e CMV assays due to their slightly smaller amplicon size compared to LDT CMV (15).

Limitations of our study include the use of surplus samples for the assay comparison, which in some cases did not provide sufficient sample volume for additional analysis. Overall, longitudinal comparison of patient courses comparing LDT and Alinity m confirmed the high correlation between the two assays.

Current routine procedure of sample collection and batchwise testing with a frequency between twice a day and once a week and onboard TATs between 3.5 and 9 h (12, 16, 17) lead to an average total TAT of 1–8 days from sample receipt in the laboratory to result reporting across the three study sites. In contrast, random and continuous access capabilities of Alinity m without the need for batching combined with a median onboard TAT of less than 3 h enable same-day reporting of actionable results which is important for transplant patient management. Similar rapid and homogenous TAT results have already been reported for other Alinity m assays (16, 17).

In conclusion, this multicenter evaluation of the newly developed real-time PCR-based Alinity m CMV assay showed high precision, reproducibility, as well as accurate quantitation of CMV using verification panels and clinical plasma samples from solid organ or hematopoietic stem cell transplant recipients. The fully automated Alinity m platform enables same-day reporting of CMV test results allowing shortening the time between diagnosis and treatment and, thus, may improve patient care and outcome.
